# Coronary artery calcification score as a prognostic factor in patients undergoing allogeneic stem cell transplantation – a retrospective analysis

**DOI:** 10.1186/s40959-026-00555-2

**Published:** 2026-09-10

**Authors:** Hans-Jonas Meyer, Anar Aghayev, Narmin Nasibova, Felix Barajas Ordonez, Jan Borggrefe, Alexey Surov

**Affiliations:** 1 Department of Diagnostic and Interventional Radiology, University of Leipzig, Leipzig, Germany; 2 University Clinic for Radiology and Nuclear Medicine, Otto- von- Guericke-University Magdeburg, Magdeburg, Germany; 3 Department of Diagnostic and Interventional Radiology, University Hospital RWTH, Aachen, Germany; 4 Department of Radiology, Neuroradiology and Nuclear Medicine, Johannes-Wesling Klinikum Minden, Ruhr-University-Bochum, Minden, Germany

**Keywords:** CT, CAC score, Hematology, Stem cell transplantation

## Abstract

**Background:**

Coronary artery calcification (CAC) scoring can be measured as a by-product of computed tomography (CT). CAC scoring reflects the general cardiovascular risk profile of patients. The aim of the present study was to determine the prognostic role of CAC on overall survival (OS) in patients with different hematological diseases undergoing allogeneic stem cell transplantation.

**Methods:**

A retrospective analysis was conducted on all patients undergoing peripheral blood stem cell transplantation between January 2015 and October 2021. A total of 121 patients (68 male patients, 56.1%) with a mean age of 53.1 ± 13.6 years were identified in the data base and included into the present study. The clinical CT scan to rule out infectious foci was used to assess the CAC score for each patient. The semiquantitative Weston score was measured to quantify CAC.

**Results:**

A total of 67 patients (54.9%) died during the course of the study. A total of 52 patients (42.6%) had visible calcifications on CT images, whereas 70 patients (57.4%) had no calcifications (CAC score 0). There was no association between the CAC score with OS, with an HR of 1.14 (95% CI 0.89; 1.45), *p* = 0.28) as a metric variable and HR of 1.33 (95% CI 0.82; 2.15) as a dichotomized variable (CAC score 0 versus positive CAC score). There was no association between CAC score and occurrence of sepsis or graft-versus host disease after transplantation.

**Conclusions:**

The presence of CT-defined coronary calcifications has no prognostic relevance in patients undergoing allogeneic stem cell therapy. The impact of CT-defined cardiovascular risk factors appears to be relatively modest in this heterogeneous patient sample.

## Introduction

Computed tomography (CT)-defined coronary artery calcification (CAC) scoring is widely used to quantify the calcified plaque burden of the coronary arteries. It is an important prognostic factor in patients with coronary artery disease [1–3]. Notably, CAC score can predict the occurrence of major cardiovascular events in asymptomatic individuals and in several patient groups throughout medicine, mainly due to the fact of a general reflection of cardiovascular health state of the individuals [3].

In general, the Agatston score is calculated on cardiac-gated CT images [1], whereas the more recently developed Weston score is calculated semiquantitatively on non-gated CT images and therefore, can be obtained from staging CT images [4, 5].

In addition to the clear published benefits of CAC scoring in cardiology and cardiovascular medicine, also promising results of the prognostic relevance in oncological patient cohorts have been published [6–10]. Especially, the prognostic implications of CAC in lung cancer patients have been explored [8, 10]. It is known that the cardiovascular risk profile of the patient is important for patients undergoing allogeneic peripheral blood stem cell transplantation (aPBSCT) due to the cardiotoxic effect of this treatment [11].

Recently, several studies demonstrated the prognostic effect for CAC in hematological patients with different results [12–16]. This clearly indicates that more scientific effort is needed to validate this imaging marker before translation into clinical routine.

The aim of the present study was to investigate the prognostic relevance of CT-derived CAC scoring for overall survival in patients undergoing aPBSCT in a single center. The hypothesis is that CAC scoring has prognostic information in this selected patient cohort with a high risk of cardiotoxicity.

## Methods

### Patient acquisition

This retrospective observational study was approved by the institutional review board (Nr. 145/21, Ethics Committee, Otto-von-Guericke University of Magdeburg, Magdeburg, Germany).

A power analysis was conducted to test for the needed number of patients. Under the general consideration of a mortality rate of 21.5% for a general patient cohort undergoing CT-imaging with a negative CAC-score and a resulting 50% increase of mortality with a positive CAC-score [17], the needed number of patients is 126 to achieve a statistical power of 80%.

All patients with different hematological diseases undergoing allogenic PBSCT were retrospectively included in the study between the years between January 2015 and October 2021. In all cases, an aPBSCT was performed in a curative intent. This patient cohort was previously investigated and published elucidating the prognostic relevance of CT-defined body composition [18].

Inclusion criteria were the following: (a) patients (≥ 18 years old) who underwent their first aPBSCT for confirmed leukemia (acute or chronic, regardless of the phenotype), MDS, or MPN (b) available CT of the chest before the transplant within four weeks prior treatment.

The exclusion criteria were as follows: (a) no available CT and (b) previous hematopoietic stem cell transplantation.

CT scans were performed in clinical routine to rule out occult infection before the initiation of conditioning therapy. Patients were identified in our internal clinical database (MEDICO KIS, CompuGroup Medical SE & Co. KGaA, Koblenz, Germany). Clinical information was extracted and comprised of gender, age, height, weight, body mass index (BMI), total serum protein (g/dL), total serum albumin (g/dL), and allo-HSCT donor source.

### Overall survival and adverse treatment effects

OS was defined as the time frame from transplant to death or the date of the last contact at the end of the observation period in February 2023. The progression-free survival (PFS) was defined from transplant to first recurrence of disease.

Early post-transplant adverse events within the initial 30 days after were retrieved from patients’ medical records and graded according to the Common Terminology Criteria for Adverse Events (CTCAE Version 4.03) of the National Institutes of Health [19]. These were defined as septic focus or graft-versus host disease.

### CT technique and CAC scoring

CT scans were performed on a Canon Aquilion Prime (Canon Medical Systems, Otawara, Japan) or Siemens SOMATOM Definition AS+ (Siemens Healthcare, Erlangen, Germany) multidetector CT scanner. The protocol was as follows: acquisition slices thickness of 1 mm with reconstructions of 5 mm, tube voltage of 120 kV, automated tube current modulation, pitch factor of 1.2., and collimation of 0.6 mm. The CT was acquired during clinical routine to rule out infectious foci before transplantation. No contrast media was applied for the CT scan.

**Fig. 1:**
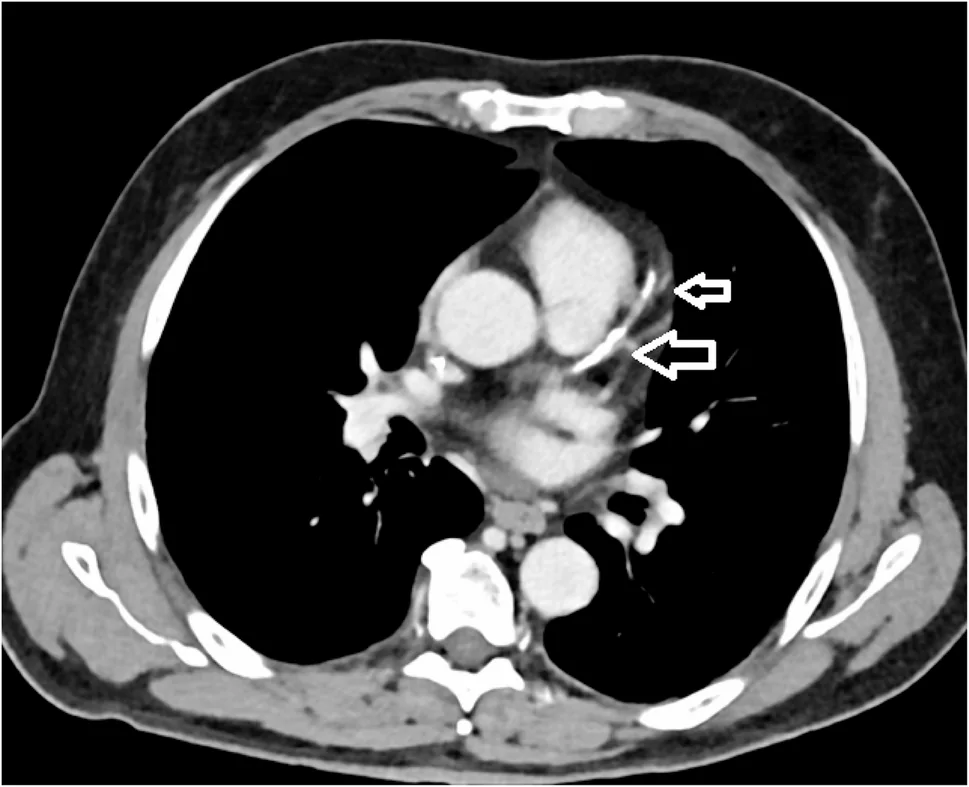
Representative 62-years old male patient of the patient sample. The arrows indicate the extensive calcifications in the left anterior descending artery with a score of 3 and of the left main coronary artery with a score of 3. The left circumflex artery and the right coronary artery were not affected. The resulting Weston score for this patient is therefore 6

The Weston scoring was performed by an experienced radiologist with seven years of experience in cardiovascular radiology. The scoring was described previously [4, 14]. In short, the score can range from 0 to 12, from no visible calcification to very severe calcification of every coronary artery. A second reader performed all measurements blinded to the first results to provide an interreader agreement. Figure 1 depicts a representative patient from the study, illustrating the CT-defined CAC score.

### Statistical analysis

The statistical analysis and graph generation were conducted using GraphPad Prism 10 (GraphPad Software, La Jolla, CA, USA) and the SPSS package (IBM SPSS Statistics for Windows, version 225.0: IBM Corporation). The data were expressed using descriptive statistics including absolute and relative frequencies. Spearman’s correlation coefficient (r) was employed to examine the associations between CAC score and clinical parameters. Intraclass coefficients (ICC) with a two-Way mixed model were used for interreader agreement. The Mann-Whitney-U test and the Fisher’s exact test were used to test for group differences when appropriate.

A binary logistic regression analysis was used for associations between CAC score and the occurrence of graft-versus host disease. Kaplan-Meier curves were used to visualize the survival times stratified to CAC score. Log-rank test was used for the survival differences of the groups. The Cox proportional hazards regression was used to analyze the associations between CAC score with the overall- and progression free survival.

In all instances, a p-value of less than 0.05 was set as the significance level.

## Results

The present analysis included 121 patients (68 male patients, 56.1%) with a mean age of 53.1 ± 13.6 years, with sufficient clinical and imaging data for the analysis.

A total of 67 patients (55.3%) died during the observation period.

The median observation period was 34 months, range 1–97 months. The median OS time was 22.62 months (IQR: 7.07–58.01 months).

The most common diagnosis was AML in 59 patients (48.7%), followed by MDS in 15 patients (12.4%) and ALL in 14 patients (11.6%). The majority of the patients received their allo-HSCT from an unrelated donor [*n* = 100 (82.6%)], followed by a matched related donor in 17 cases (14.0%). Table 1 summarizes the demographics of the patient sample.

**Table 1:** Demographic overview of the investigated patient sample

Parameter	*N* = 121 (%)
Age (years) Median (IQR)	57 (44;64)
female	53 (43.9%)
Visible calcification	52 (42.6%)
Diagnosis	
Acute myeloid leukemia (AML)	59 (48.7)
Acute lymphoblastic leukemia (ALL)	8 (6.6)
Mixed-phenotype acute leukemia (MPAL)	4 (3.3)
Chronic lymphocytic leukemia (CLL)	12 (9.9)
Myeloproliferative neoplasms (MPN)	5 (4.1)
Myelodysplastic syndrome (MDS)	20 (16.3)
Others	13 (11.7

The mean CAC score was 0.7 ± 0.95, median 0, ranging from 0 to 3. The interreader agreement for the CAC score was excellent with an ICC of 0.95 (95% CI 0.93;0.97).

**Fig. 2:**
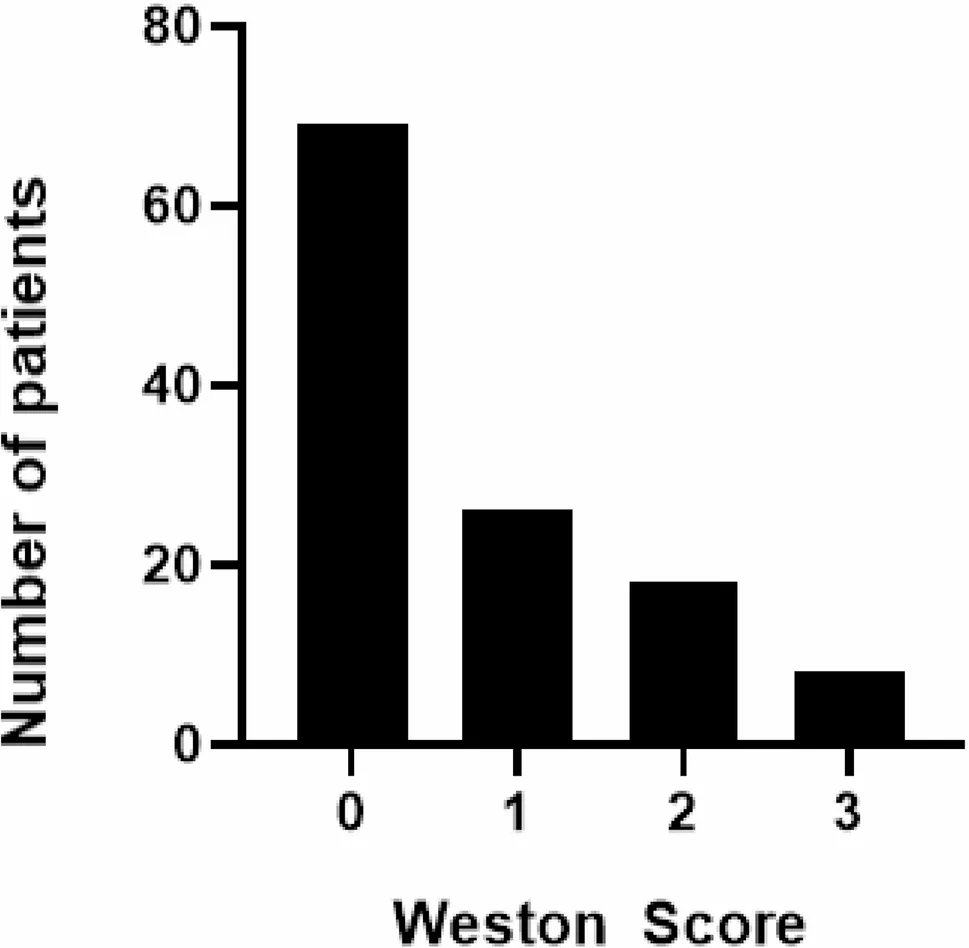
Histogram distribution of the CAC score of the patient cohort. A total of 52 patients (42.6%) had visible calcifications on CT images, whereas 70 patients (57.4%) had no calcifications (CAC score 0)

**Fig. 3:**
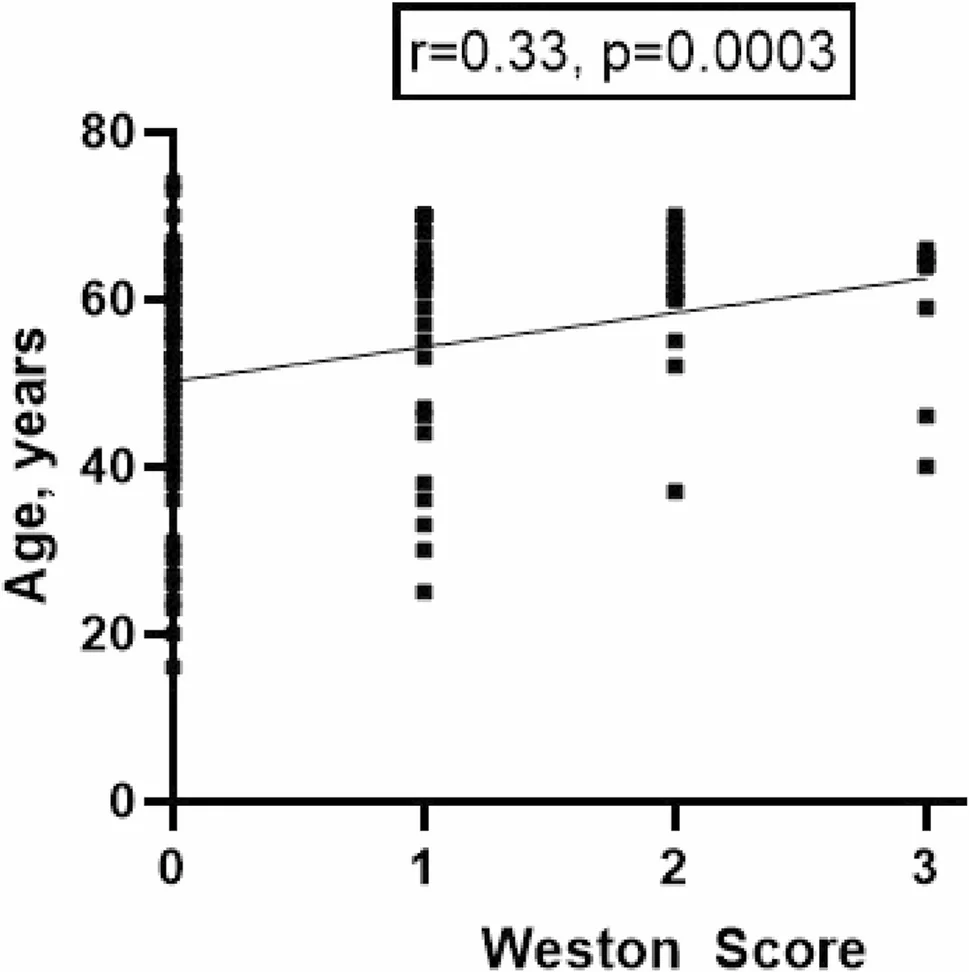
Spearman’s correlation analysis between the CAC score with age (*r* = 0.33, *p* = 0.0003)

A total of 52 patients (42.6%) had visible calcifications on CT images, whereas 70 patients (57.4%) had no calcifications (CAC score 0). Figure 2 provides the CAC score distribution of the patient cohort. There was a moderate correlation between CAC score with age (*r* = 0.33, *p* = 0.0003, Fig. 3).

**Fig. 4:**
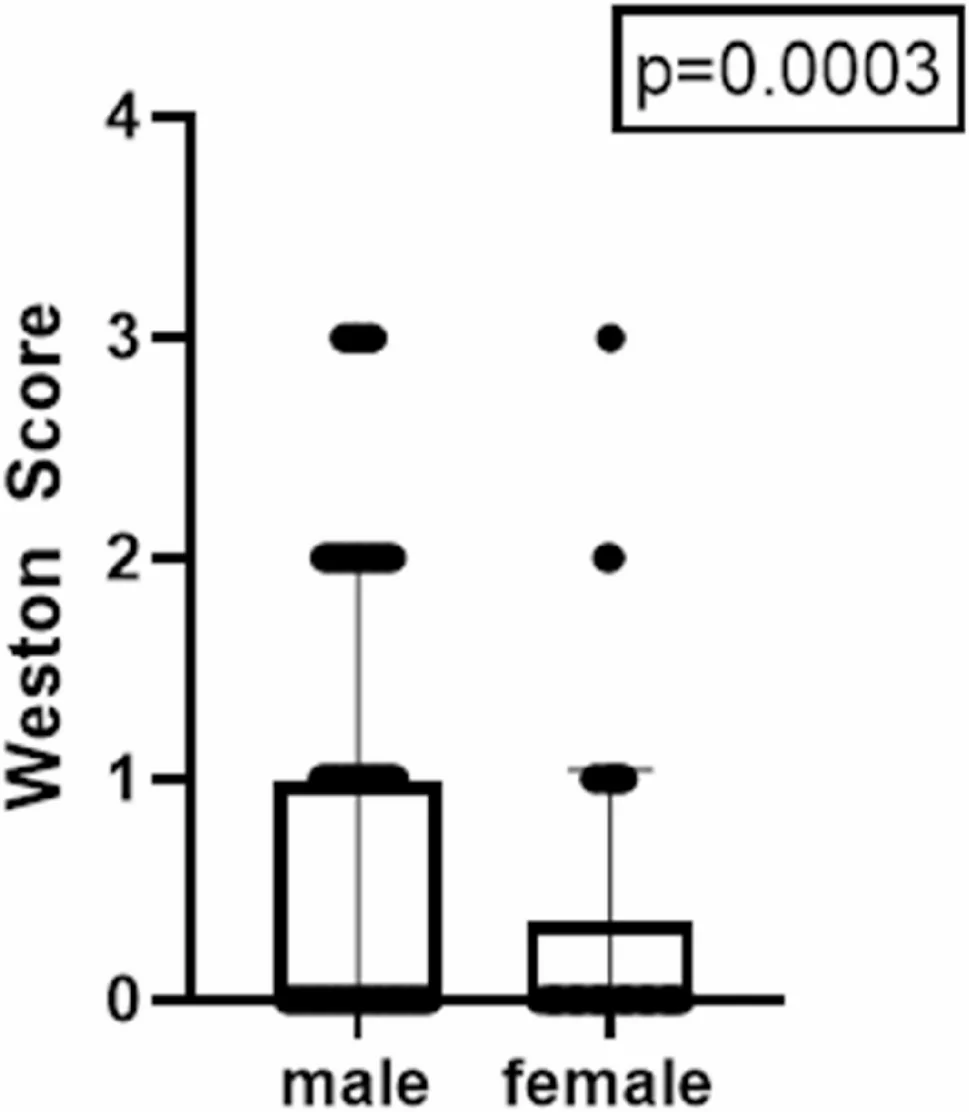
Comparison between the CAC score of the male and female patients in this patient cohort. Male patients had significantly higher CAC values compared to female patients (*p* = 0.0003). The whiskers represent the interquartile range

The CAC score was significantly higher in male compared to female patients (median 1 versus 0, *p* = 0.0003, Fig. 4).

## Overall survival and progression-free survival

The CAC score did not differ between non-survivors and survivors in discrimination analysis (mean 0.77 ± 0.95 versus 0.64 ± 0.95, *p* = 0.37).

In the lethal group, there were 32 patients with a positive CAC score (47.1%) and in the survivor group there were 21 patients (38.8%). This ratio was not different (*p* = 0.61).

There was no association between the CAC score with OS, with an HR of 1.14 (95% CI 0.89; 1.45), *p* = 0.28) as a metric variable and HR of 1.33 (95% CI 0.82; 2.15) as a dichotomized variable (CAC score 0 versus positive CAC score) (Table 2).

**Table 2:** Cox regression analysis for the effect of the investigated CAC parameters on OS (Univariable Analysis)

Parameters	HR	95%CI	*p*-values
Weston Score metric	1.14	(0.89, 1.45)	0.28
Weston Score (positive)	1.33	(0.82, 2.15)	0.24
Sex (1 = male)	0.75	(0.46, 1.21)	0.08
Age (metric)	1.02	(1.0, 1.04)	0.05

*Abbreviations*: *HR * Hazard ratio, *CI* Confidence interval

In a multivariable analysis adjusted for age, sex, occurrence of graft-versus host disease and tumor entity, the results were HR of 1.20 (95% CI 0.89; 1.61), *p* = 0.22 as a metric variable and HR of 1.35 (95% CI 0.77; 2.39), *p* = 0.28 as a dichotomized variable.

Similar results were identified for the associations between CAC score with PFS.

The HR was 0.98 (95%CI 0.69; 1.38, *p* = 0.91) as a metric variable and HR of 1.43 (95% CI 0.75; 2.71, *p* = 0.26) as a dichotomized variable.

In a multivariable analysis adjusted for age, sex, occurrence of graft-versus host disease and tumor entity, the results were HR of 0.93 (95% CI 0.63; 1.38), *p* = 0.73 as a metric variable and HR of 1.41 (95% CI 0.69; 2.90), *p* = 0.33 as a dichotomized variable.

**Fig. 5:**
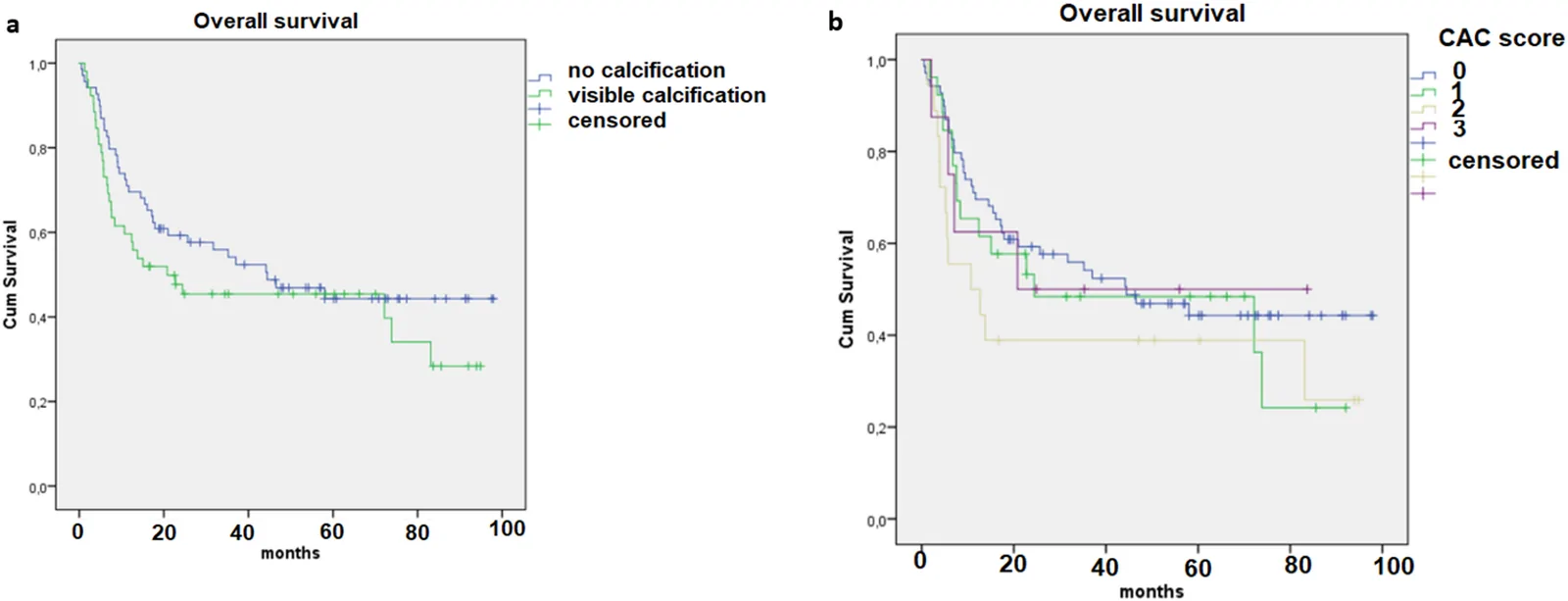
**A** Kaplan-Meier curves for the overall survival comparison between patients without calcifications (CAC score 0) and with calcifications. The median survival for the group without calcifications (CAC score of 0) was 44.4 months, compared to the group with calcifications was 20.8 months, Log rank test *p* = 0.24, Wilcoxon test *p* = 0.18. **B** Kaplan-Meier curves with stratifications for calcification groups according to the CAC score (Log rank test *p* = 0.47, Wilcoxon test *p* = 0.28)

In the Kaplan-Meier curves, similar results are provided (Fig. 5). The median survival for the group without calcifications (CAC score of 0) was 44.4 months, compared to the group with calcifications was 20.8 months, Log rank test *p* = 0.24, Wilcoxon test *p* = 0.18 (Fig. 5a). With consideration of CAC as a metric valuable, there was also no statistically significant difference between the groups (Log rank test *p* = 0.47, Wilcoxon test *p* = 0.28 Fig. 5b).

### Associations with complications

Overall, 52 patients developed graft-versus host disease (42.9%) after the transplantation. There was no association with CAC score as a metric (OR 0.96, 95% CI 0.69; 1.41, *p* = 0.85) or dichotomized variable (OR 0.95, 95% CI 0.46; 1.97, *p* = 0.89). Similarly, there was no association with transplantation-associated sepsis, which occurred in overall 20 patients (16.5%). The resulting OR was 1.25 (95% CI 0.78; 2.03, *p* = 0.33) for the metric variable and OR of 1.40 (95% CI 0.53; 3.67, *p* = 0.48) for the dichotomized variable.

## Discussion

The present study demonstrates that CT-defined CAC score is not a prognostic factor in a heterogenous patient sample undergoing allogeneic PBSCT. This was shown for the sole presence of coronary artery calcification as well as for the Weston score as a semiquantitative marker.

Hypothetically, CAC scoring may be important in patients undergoing PBSCT as there is strong evidence for the cardiotoxicity induced by this form of treatment, which can be stratified by short term effects caused by treatment conditioning regimen and graft-versus host effects and then by long term effects with a risk profile for coronary artery disease [11, 20, 21]. The reported incidence of acute heart failure after PBSCT ranges from 0.4 to 2.2% [21].

CT-defined CAC scoring is an extensively studied imaging biomarker, most prominently investigated within the Framingham Heart study [2, 3, 22]. Since then, the prognostic significance of coronary artery calcified plaque burden in patients with coronary artery disease has been clearly established and translated into clinical practice as a relevant imaging for general vascular status [1–3].

Until recently, CAC scoring was only performed on cardiac-gated CT images with the Agatston method with therefore little general translation for standard CT images acquired during clinical routine [1]. The Weston score used in the present study is highly correlated and reliable when compared to the Agatston score and be considered as a good surrogate score [4, 5].

Nevertheless, despite its high prevalence in clinically asymptomatic patients and the high frequency of staging CT investigations, the prognostic relevance of CAC scoring remains a topic that has only been explored in a limited number of studies within the field of oncology.

A recent PET-CT study investigating a heterogenous oncological patient sample with solid tumors, CAC was associated with the primary end point (a composite of death and cardiovascular) after adjustment in multivariable analysis (OR 2.6 (95% CI 1.42–4.77) (*p* < 0.01) [23].

First studies could also show the prognostic relevance in the field of hematology [12–16, 24]. Among them, a significant multicenter study on diffuse large B-cell lymphoma, the CAC score was automatically measured in 1,468 patients and was highly associated with cancer therapy-related cardiac dysfunction and major adverse cardiovascular events [24]. This information could provide clinicians further information on the cardiovascular risk profile of patients at risk receiving anthracycline-based chemotherapy.

In another study investigating chronic myeloid leukemia, CAC score was associated with a higher cardiac risk [25].

The present study corroborates a recent study investigating patients with multiple myeloma undergoing autologous stem cell transplantation [14]. In this mentioned study, there was also no significant association between the Weston score and overall and progression free survival after stem cell transplantation. However, the patients in this mentioned study had more visible calcification resulting in a higher Weston score despite similar mean ages of the patient samples.

One important aspect is the distinctive differences between autologous and allogenic stem cell transplantation [26, 27]. Important drivers for early arteriosclerosis after autologous stem cell transplantation is the graft-vs.-host disease, which is mediated by CD8 positive T-cells [27].

Our findings revealed a higher prevalence of coronary calcifications in male patients compared to the female patients, which is a known finding throughout the literature [1–4]. This also strengthens the fact to take gender differences of CAC scoring into account. However, this did not result in significant differences in survival analysis despite adjustment of gender. One can only deduct that the observed higher cardiovascular risk profile in the investigated patient sample for the male patients did not result in a detectable survival outcome. Presumably, this was adjusted for during clinical routine.

In a recent study on a more selected patient cohort undergoing post-transplant cyclophosphamide therapy after stem cell transplantation, positive CAC score was not related to OS in the whole cohort, but among elderly patients age over 60 years and without a history of coronary artery disease, CAC score was associated with an inferior 2-yr OS (HR 3.67, 95% CI 1.007–13.38, *P* = 0.049) [12].

Clearly, there is more research needed in the field of cardio-oncology to elucidate the complex interactions between CAC score as a cardiovascular risk imaging marker and survival outcomes in haematological diseases. Exact age and sex stratifications based on larger multicentric patient samples will be needed to move the field forward.

A potential cause for the present negative results could also be that the mortality after stem cell transplantation may be primarily driven by other factors than the general cardiovascular risk profiling. The risk for infections, relapse of the disease, treatment complication can be of higher relevance than the overall risk for cardiovascular events in this patient cohort.

It remains of great importance to better characterize the general cardiovascular risk profile of patients undergoing hematopoietic stem cell transplantation as the general prevalence of cardiovascular diseases is 16.8% in the general population with distinctive differences in subgroup analyses [28].

It should definitely be emphasized that a potential prognostic impact of the CAC scoring on the survival outcomes may be obscured by the small size and heterogeneity of the patient sample. The present analysis can therefore only provide first results that the overall prognostic effect of CT-defined CAC scoring may be low and, in most cases, not clinically relevant. However, the completely negative results of the present analysis should be discussed with care.

There are limitations of the present study to address. First, the study population encompassed a broad spectrum of hematological malignancies, potentially introducing heterogenicity into the analysis. The role of comorbidities or previous treatments, or adjuvant therapy as confounders was not explored. Additionally, the retrospective methodology and the monocentric setting limit the generalizability of our findings. Moreover, a potential selection bias has to be discussed. The mean CAC scores in the analyzed patient cohort is relatively low with lack of patients with severe score values. This could be explained by a potential exclusion of patients with a high cardiovascular risk profile of the stem cell therapy beforehand. It remains unclear, whether the CT-defined CAC score has a potential prognostic role in those patients at high risk for cardiovascular events. Second, CAC scoring was conducted using a non-cardiac gated CT. However, there is sufficient data that the Weston score used has a very high correlation with the gold standard Agatston score (*r* = 0.83) [4].

Third, there is a low risk for loss to follow-up of the patients. However, the high frequency of the mortality events of the present cohorts demonstrates a low risk of bias in the overall survival analysis. Fourth, there is lack of clinical and anamnestic information of the patient sample. The CT-defined CAC scoring has only a clinical benefit, if it provides more information of the patients than the general cardiovascular risk profiling using anamnestic information alone. This needs to be addressed by future studies. Moreover, there is need for further investigations of the occurrence of major cardiovascular events after stem cell transplantation stratified by CT-defined CAC scoring. This could not be performed in the current study due to lack of clinical information. Fifth, there is a potential risk for type II errors due to our small patient sample. The present results should therefore be read under the potential of lack of statistical power. A larger, multicentric data set could potentially show a small prognostic role of CAC scoring in patients undergoing allogenic stem cell transplantation in the future.

## Conclusions

The presence of CT-defined coronary calcifications seems to have no clinically relevant prognostic role for survival outcomes in patients undergoing allogeneic stem cell therapy. The impact of CT-defined cardiovascular risk factors appears to be relatively limited in this heterogeneous patient cohort, despite their high prevalence, observed in 42.6% of patients. Larger patient cohorts are needed to show a potential impact of CT-defined coronary calcification scoring on the survival outcomes which could be falsely not detected in the present cohort.

## Data Availability

No datasets were generated or analysed during the current study.

## Abbreviations

aPBSCT: allogeneic peripheral blood stem cell transplantation
CT: Computed tomography
CI: Confidence interval
CAC: Coronary artery calcification
HR: Hazard ratio
MM: Multiple myeloma
OS: Overall survival
PFS: Progression free survival
